# Dietary habits and self-reported hypoxic events in competitive freedivers: an observational study

**DOI:** 10.3389/fnut.2026.1849515

**Published:** 2026-07-07

**Authors:** Jordi Sarola-Gassiot, Alfredo Irurtia, Ishar Dalmau-Santamaria, Lara Rodríguez-Zamora

**Affiliations:** 1 National Institute of Physical Education of Catalonia (INEFC), University of Barcelona (UB), Barcelona, Spain; 2 Health Department, Fundació TecnoCampus Mataró-Maresme, Research Group in Hypoxia, Dietetics, Nutrition and Kinanthropometry (ReGHyDiNuK), Mataró, Barcelona, Spain; 3 INEFC-Barcelona Research Group on Sport Sciences (GRCEIB), National Institute of Physical Education of Catalonia (INEFC), University of Barcelona (UB), Barcelona, Spain; 4 School of Health and Medical Sciences, Division of Sport Sciences, Örebro University, Örebro, Sweden

**Keywords:** apnea, athlete safety, loss of consciousness, diving, hypoxia, sports nutrition, stimulant beverages

## Abstract

**Introduction:**

Competitive freediving involves repeated voluntary breath-holding, during which hypoxic events such as loss of motor control (LMC) or blackout (BO) may occur. Although nutrition may influence metabolic responses to hypoxia, evidence in competitive freedivers remains limited. This study aimed to investigate the relationship between dietary habits and self-reported hypoxic events in Spanish competitive freedivers.

**Methods:**

A cross-sectional study was conducted in 71 competitive freedivers (19 women: 39.4 ± 9.3 years; 52 men: 41.9 ± 8.6 years). Participants completed a questionnaire on training characteristics and hypoxic events. Events were classified as LMC or BO, and participants were categorized according to the most severe event experienced. Dietary habits were assessed using a validated food frequency questionnaire (PREDIMED). Associations were examined using chi-square or Fisher’s exact tests, and significant variables were included in a binomial logistic regression adjusted for age, sex, and years of practice.

**Results:**

Overall, participants showed moderate consumption across dietary groups, with higher intake of fruits, vegetables, and protein-rich foods; and low intake of ultra-processed foods and alcohol. Male divers consumed moderate amounts of protein-rich foods, whereas female counterparts reported lower intake [*χ^2^*(2) = 8.53, *p* = 0.014; Fisher’s exact test, *p* = 0.010]. Overall, 21 freedivers (29.6%) reported at least one hypoxic event. The occurrence of hypoxic events were associated with years of practice (*p* = 0.003) and consumption of stimulant beverages (*p* = 0.013). Beginners experienced fewer events than expected, whereas divers with >4 to 10 years of practice were overrepresented. In the adjusted model, years of practice remained significantly associated with hypoxic events [OR = 3.06, 95% CI (1.43, 6.53), *p* = 0.004]. Moderate stimulant beverage consumption emerged as a potential associated factor [OR = 12.54, 95% CI (1.27, 123.9), *p* = 0.031].

**Conclusion:**

Hypoxic events in competitive freedivers were primarily associated with years of practice, suggesting that cumulative exposure may be a relevant risk factor. Overall dietary habits showed no clear relationship with hypoxic events, although stimulant beverage consumption may warrant further investigation.

## 1 Introduction

Competitive freediving is an underwater sport based on voluntary breath-holding (apnea), practiced in both pool and open-water environments. Disciplines include static apnea (STA), in which divers remain motionless while holding their breath (1), and dynamic apnea performed horizontally in a pool with or without fins (2). In open-water competitions, divers perform depth disciplines such as constant weight, free immersion, and variable weight (3). Despite differences in movement patterns and environmental conditions, all freediving disciplines involve prolonged breath-holding, exposing athletes to substantial hypoxic stress. Factors such as a limited amount of oxygen, hydrostatic pressure, and the altered physical properties of water impose significant physiological demands on the diver.

These conditions entail unique medical risks. While other aquatic sports–such as underwater hockey, underwater rugby, or artistic swimming–also involve breath-holding, freedivers are particularly exposed to severe hypoxia and hypercapnia during maximal apnea efforts. This risk is further exacerbated in deep diving, where rapidly changing ambient pressure adds an additional layer of physiological stress. Consequently, freediving can result in various medical complications; with severe hypoxia representing the key risk factor. Hypoxic events typically manifest as loss of motor control (LMC, “samba”) or hypoxic blackout (BO), which constitute the most severe neurological consequences of cerebral hypoxia (4). LMC is characterized by involuntary motor disturbances with preserved consciousness, whereas BO involves a transient loss of consciousness (4). Although these events are well recognized, the physiological mechanisms underlying their occurrence remain incompletely understood (5, 6).

Hypoxic events are not merely theoretical; competitive freediving is associated with a measurable incidence of hypoxic episodes. Observations from the 4th AIDA World Free Diving Championship reported 35 hypoxic events among 57 participants, including several cases of BO (7). Analyses of official competition data further indicate that approximately 9.7–11.1% of performances are affected by cerebral hypoxia, depending on the discipline (8).

Given the clinical relevance of hypoxic events, identifying modifiable lifestyle factors–such as nutrition–may provide insights into strategies to mitigate risk and optimize performance. Previous research has primarily focused on acute nutritional strategies aimed at enhancing apnea performance, including pre-apnea fasting and nitrate supplementation (9, 10).

Experimental evidence suggests that fasting may prolong static apnea duration, potentially through metabolism–limiting mechanisms and an enhanced diving response (9). This interpretation is consistent with evidence showing that caloric restriction can reduce resting metabolic rate through adaptive thermogenic mechanisms (11, 12).

Similarly, nitrate-rich supplementation has been investigated for its potential effects on oxygen utilization, and cardiovascular responses during breath-hold diving (10). In contrast, higher carbohydrate availability may be associated with increased metabolic demand, which could accelerate oxygen consumption and potentially reduce apnea duration (13).

However, existing studies have primarily focused on short-term nutritional interventions and their effects on acute apnea performance, while the potential role of habitual dietary patterns remains largely unexplored in competitive freedivers. Recent expert recommendations suggest that proper nutrition and healthy eating habits may support energy balance, acid–base regulation, and recovery (14). In addition, evidence from cardiovascular research indicates that adherence to healthy dietary patterns, such as the Mediterranean diet, is associated with improved cardiometabolic health and reduced cardiovascular risk (15). These adaptations may be particularly relevant in apnea disciplines, where cardiovascular efficiency and metabolic regulation are critical determinants of performance and safety under hypoxic stress (2).

In this context, habitual dietary patterns may also contribute to hypoxia tolerance through several physiological mechanisms. Long-term dietary habits can influence metabolic flexibility, mitochondrial efficiency (16), and substrate utilization (17), all of which are key determinants of oxygen consumption during apnea. Diets rich in antioxidants and anti-inflammatory components–such as those characteristic of Mediterranean dietary patterns–may improve endothelial function and reduce oxidative stress (18), potentially enhancing tolerance to hypoxic conditions. Furthermore, the long-term distribution of macronutrient intake (i.e., carbohydrates, proteins, and fats) may influence basal metabolic rate and overall oxygen demand, which may in turn affect hypoxia tolerance.

Given the increasing popularity of competitive freediving and the potential implications for athlete safety, further research is needed to better understand lifestyle factors that may contribute to the occurrence of hypoxic events. Therefore, the primary aim of this study was to investigate the relationship between dietary habits and the occurrence of self-reported hypoxic events in Spanish competitive freedivers, while secondary analyses examined the role of gender and years of practice. We hypothesized that specific dietary habits–including fasting, carbohydrate intake, stimulant beverage consumption, and alcohol use–may be associated with the occurrence of hypoxic events during the sporting careers of competitive freedivers.

## 2 Method

### 2.1 Study design and participants

A cross-sectional observational study was conducted among competitive freedivers holding an official federation license in Spain. Participants were recruited through Spanish freediving clubs. A total of 71 divers participated in the study, including 19 women (age 39.4 ± 9.3 years); and 52 men (age 41.9 ± 8.6 years). Divers had varying years of experience and trained in different freediving disciplines, both in pools and in open-water depth environments. Inclusion criteria required participants to have competed in freediving events within the previous year, to be free from injury, and to possess a valid medical clearance for competition. All participants provided written informed consent prior to participation. The main characteristics of the participants are presented in Table 1.

**Table 1:** Anthropometric and sport-related characteristics of competitive freedivers by sex.

General characteristics		Women (*n* = 19)	Men (*n* = 52)	*p-*value
	Age (years)	39.4 ± 9.3	41.9 ± 8.6	
	Height (cm)	166 ± 6.8*	179.6 ± 8.3	<0.001
	Body weight (kg)	59.8 ± 7.4*	77.8 ± 10.5	<0.001
	BMI (kg/m^2^)	21.7 ± 2.2*	24.2 ± 3.1	<0.001
	Personal best in STA (s)	285.4 ± 71.7	325.1 ± 93.9	
**Years of practice**				<0.001
	1 year	11 (57.9%)^†^	6 (11.5%)^†^	
	> 1 to 4 years	4 (21.1%)	15 (28.8%)	
	> 4 to 10 years	4 (21.1%)	19 (36.5%)	
	> 10 years	0^†^	12 (23.1%)^†^	
**Freediving disciplines** ^§^				
	Pool disciplines	15 (78.9%)	40 (76.9%)	
	Depth disciplines	7 (36.8%)	31 (59.6%)	

Data are presented as mean ± standard deviation (SD) for continuous variables, and number of participants (*n*) and percentage (%) for categorical variables. Percentages are calculated within each gender. ^**§**^ Participants could be classified in more than one freediving discipline. BMI, body mass index; STA, static apnea. ^*^p < 0.05 vs. men. ^†^Cells contributing significantly to the association according to adjusted standardized residuals (|residual| ≥ 1.96).

### 2.2 Procedures

Data were collected through a purpose-designed online questionnaire that was self-administered from 26 March 2023 to 13 May 2023, and included sections on demographic information, characteristics of sports participation, history of hypoxic events during the athletic career, and dietary habits. Sport-related information included years of practice [≤ 1 year (beginners), > 1 to 4 years, > 4 to 10 years, and > 10 years)], competitive level (amateur, national, and international), and the type of freediving disciplines practiced (pool and depth disciplines) (2, 3). All participants practiced STA, and their personal best performance in STA is presented in Table 1.

### 2.3 Measurements

#### 2.3.1 Dietary habits

Dietary habits were assessed using a validated semi-quantitative food frequency questionnaire (FFQ) developed for the PREDIMED study and previously validated in a Spanish Mediterranean population (19, 20). The instrument, derived from the Willett FFQ and previously validated in Spain, includes 140 food items and questions on vitamin and mineral supplement use. Participants reported their habitual intake using ordinal frequency categories. Food items were grouped into major dietary categories, including dairy products, protein-rich foods, fruit and vegetables, legumes and cereals, oils and fats, ultra-processed foods, non-alcoholic beverages, stimulant beverages (e.g., coffee and tea), and alcoholic beverages. For each category, a consumption score was calculated as the mean frequency of intake of the items included in that group. To facilitate statistical analyses, scores were categorized into tertiles representing low, moderate, and high consumption.

#### 2.3.2 Occurrence of hypoxic events

Participants reported whether they had experienced hypoxic events during their sporting career through the study questionnaire. Events were self-reported and classified into two categories based on commonly used terminology in freediving: LMC and BO as previously described in the introduction. When more than one event had occurred, participants were classified according to the most severe event reported. For statistical analyses, participants were also categorized using a binary variable indicating the presence or absence of any hypoxic events.

### 2.4 Statistical analysis

Statistical analyses were performed using SPSS (version 28.0; IBM Corp., Armonk, NY, United States). Descriptive statistics were calculated for all variables. Continuous variables are presented as mean ± standard deviation (SD), while categorical variables are reported as frequencies and percentages. Normality of continuous variables was assessed using the Shapiro–Wilk test. Comparisons between men and women were performed using independent-samples t-tests or Mann–Whitney U tests when normality assumptions were not met. To compare continuous variables across the four experience groups, one-way ANOVA or Kruskal–Wallis tests were applied as appropriate according to distributional assumptions. Associations between categorical variables and the occurrence of hypoxic events (LMC and BO) were examined using chi-square (χ^2^) tests or Fisher’s exact tests when expected cell counts were <5. When significant associations were identified, adjusted standardized residuals were examined to determine which cells contributed most to the association. Residuals with an absolute value >1.96 were considered statistically significant. Variables showing significant associations in the bivariate analyses were subsequently included in a binomial logistic regression model to identify independent predictors of hypoxic events, adjusting for age, sex, and years of practice. Covariates were selected based on their theoretical relevance and potential role as confounding variables, as supported by previous literature. Model diagnostics were performed to assess goodness-of-fit and the absence of multicollinearity among predictors (e.g., variance inflation factors). Statistical significance was set at *p* < 0.05.

## 3 Results

### 3.1 Description of the responders

When comparing sexes, male divers were significantly heavier (77.8 ± 10.5 *vs.* 59.8 ± 7.4 kg, *p* < 0.001) and taller (179.6 ± 8.3 *vs.* 166 ± 6.8 cm, *p* < 0.001) than female divers, resulting in a higher BMI (24.2 ± 3.1 *vs.* 21.7 ± 2.2 kg·m^−2^, *p* < 0.001; Table 1). No significant sex differences were observed in STA personal best (men: 325.1 ± 93.9 *vs.* women: 285.4 ± 71.7 s, *p* = 0.142).

A significant association was observed between sex and years of practice [*χ^2^* (3) = 18.2, *p* < 0.001; Fisher’s exact *p* < 0.001]. Analysis of standardized residuals indicated that male beginners were underrepresented in the sample (residual = −4.05), whereas those with more than 10 years of practice were overrepresented (residual = 2.30). In contrast, no significant sex differences were observed in the distribution of competitive level (*p* > 0.05), nor in the proportion of divers practicing pool disciplines [*χ^2^*(1) = 0.03, *p* = 0.857; Fisher’s exact *p* = 1.000] or depth disciplines [*χ^2^*(1) = 2.90, *p* = 0.09].

### 3.2 Dietary habits

Descriptive distributions of dietary consumption categories according to sex are presented in Table 2. Overall, most divers were classified in the moderate consumption category across the majority of food groups. Moderate-to-high consumption was most frequently observed for fruit and vegetables and protein-rich foods, whereas high consumption of ultra-processed foods and alcohol was uncommon in this cohort.

**Table 2:** Distribution of dietary consumption categories according to sex.

Food group	Sex	Low consumption	Moderate consumption	High consumption	*p*-value
Fruit and vegetables	M	13 (25.0%)	32 (61.5%)	7 (13.5%)	
	W	3 (15.8%)	10 (52.6%)	7 (31.6%)	
Legumes and cereals	M	12 (23.1%)	36 (69.2%)	4 (7.7%)	
	W	9 (47.4%)	9 (47.4%)	1 (5.3%)	
Protein-rich foods	M	10 (19.2%)^†^	38 (73.1%)^†^	4 (7.7%)	0.010
	W	10 (52.6%)^†^	7 (36.8%)^†^	2 (10.5%)	
Dairy products	M	27 (51.9%)	19 (36.5%)	6 (11.5%)	
	W	16 (84.2%)	3 (15.8%)	0 (0.0%)	
Oils and fats	M	31 (59.6%)	18 (34.6%)	3 (5.8%)	
	W	12 (63.2%)	7 (36.8%)	0 (0.0%)	
Ultra-processed foods	M	31 (59.6%)	18 (34.6%)	3 (5.8%)	
	W	14 (73.7%)	3 (15.8%)	2 (10.5%)	
Non-alcoholic beverages	M	30 (57.7%)	19 (36.5%)	3 (5.8%)	
	W	9 (47.4%)	5 (26.3%)	5 (26.3%)	
Stimulant beverages	M	20 (38.5%)	22 (42.3%)	10 (19.2%)	
	W	5 (26.3%)	10 (52.6%)	4 (21.1%)	
Alcoholic beverages	M	50 (96.2%)	2 (3.8%)	0 (0.0%)	
	W	17 (89.5%)	1 (5.3%)	1 (5.3%)	

Values are expressed as number of participants and percentage within sex. M = men, W = women. Consumption categories correspond to tertiles (low, moderate, high) derived from the frequency of intake reported in the food frequency questionnaire. Differences between men and women were assessed, and statistical significance was set at *p* < 0.05. ^†^Cells contributing significantly to the association between sex and dietary category according to adjusted standardized residuals (|residual| ≥ 1.96).

A significant association was observed between sex and protein-rich food consumption [*χ^2^*(2) = 8.53, *p* = 0.014; Fisher’s exact *p* = 0.010]. Standardized residual analysis showed that female divers were overrepresented among low consumers (residual = 2.77), whereas male divers were overrepresented among moderate consumers (residual = 2.81). No significant differences between sexes were observed in the proportion of divers practicing fasting [*χ^2^*(1) = 0.04, *p* = 0.844].

Additionally, no significant differences were found among the different years of practice groups for any of the major dietary categories (*p* > 0.05). Similarly, no significant association was found between fasting practices and years of practice [*χ^2^*(3) = 1.61, *p* = 0.658].

### 3.3 Occurrence of hypoxic events

Overall, 21 divers (29.6%) reported having experienced at least one hypoxic event during their sporting career. Among men, 16 of 52 divers (30.8%) reported a hypoxic event, including 1 case of LMC and 15 cases of BO. Among women, 5 of 19 divers (26.3%) reported hypoxic events, including 1 case of LMC and 4 cases of BO.

No significant association was found between the occurrence of hypoxic events and sex [*χ^2^*(1) = 0.133, *p* = 0.716]. However, hypoxic events were significantly associated with years of practice [*χ^2^*(3) = 11.8, *p* = 0.008; Fisher’s exact test *p* = 0.003]. Standardized residual analysis indicated that beginners showed fewer hypoxic events than expected (residual = −3.06), whereas divers with >4 to 10 years of practice showed more hypoxic events than expected (residual = 2.33).

When the type of hypoxic event was considered (none, LMC or BO), no association with sex was observed [*χ^2^*(3) = 1.152, *p* = 0.764; Fisher’s exact test *p* = 0.81]. However, a significant association was found with years of practice [*χ^2^*(6) = 19.2, *p* = 0.004; Fisher’s exact test *p* = 0.001, Table 3]. Residual analysis indicated that beginners reported fewer BO than expected (residual = −2.86), whereas divers with >1 to 4 years of practice showed more cases of LMC (residual = 2.37) and those with >4 to 10 years of practice showed more BO events (residual = 2.78).

**Table 3:** Self-reported hypoxic events according to years of freediving practice.

Years of practice	No event	LMC	Blackout
≤ 1 year	17 (100) ^†^	0	0 ^†^
> 1 to 4 years	14 (73.7)	2 (10.5)^†^	3 (15.8)
> 4 to 10 years	12 (52.2)^†^	0	11(47.8)^†^
> 10 years	7 (58.3)	0	5 (41.7)
Total	50 (70.4)	2(2.8)	19(26.8)

Data are presented as *n* (% within years-of-practice category). Associations were tested using Fisher’s exact test. LMC: loss of motor control. ^†^Cells contributing significantly to the association according to adjusted standardized residuals (|residual| ≥ 1.96). Significant association between years of practice and hypoxic events (*p* < 0.001). Statistical significance was set at *p* < 0.05.

### 3.4 Dietary habits and the occurrence of hypoxic events

A significant association was found between stimulant beverage consumption and the occurrence of hypoxic events [*χ^2^*(2) = 9.08, *p* = 0.01; Fisher’s exact test *p* = 0.013]. *Post hoc* examination of standardized residuals revealed that divers with high stimulant beverage consumption were underrepresented among hypoxic events (residual = −2.05), whereas those with moderate consumption were overrepresented (residual = 2.89).

A binary logistic regression was conducted to examine the effect of stimulant beverage consumption on the likelihood of hypoxic events, controlling for age, sex, and years of practice. The model was statistically significant, explaining 35.6% of the variance in hypoxic events (Nagelkerke *R^2^* = 0.356; Cox & Snell *R^2^* = 0.250). Years of practice was significantly associated with hypoxic events, with each increase in category associated with a higher likelihood of reporting an event [OR = 3.06, 95% CI (1.43, 6.53), *p* = 0.004]. Regarding stimulant consumption, and using the high consumption group as reference, moderate consumption was associated with higher odds of hypoxic events [OR = 12.54, 95% CI (1.27, 123.9), *p* = 0.031], whereas no significant differences were observed for low consumption [OR = 3.33, 95% CI (0.308, 35.88), *p* = 0.322]. Age and sex were not significantly associated with hypoxic events (*p* > 0.05).

## 4 Discussion

The present study aimed to investigate the relationship between dietary habits and the occurrence of hypoxic events in competitive Spanish freedivers. The main findings indicate that hypoxic events are relatively frequent, affecting nearly one-third of the sample, and are primarily associated with years of practice. In addition, stimulant beverage consumption emerged as a factor associated with hypoxic events, showing a non-linear relationship, with moderate consumption linked to higher odds of reporting such events. In contrast, no clear associations were observed between hypoxic events and overall dietary patterns or fasting practices, despite a generally moderate-to-high consumption of nutrient-dense foods within the sample. Taken together, these findings suggest that hypoxic events are more closely related to exposure and training-related factors, while specific dietary behaviors, such as stimulant consumption, may also play a role.

### 4.1 Dietary habits

The dietary profile observed in our sample of competitive freedivers was generally characterized by moderate intake across most food groups, with relatively frequent consumption of fruit and vegetables and limited representation of high intake of ultra-processed foods and alcoholic beverages. Although adherence to the Mediterranean diet was not formally assessed, this overall pattern appears broadly compatible with the Mediterranean-oriented dietary framework from which the questionnaire was derived and with recent nutritional recommendations proposed for freedivers (14). Such dietary characteristics may be relevant in apnea sports, as they have been proposed to support cardiovascular function, oxidative stress management, acid–base balance, and post-exercise recovery (14).

The only dietary difference observed between sexes concerned the consumption of protein-rich foods, with men reporting a higher habitual consumption frequency than women. Notably, female divers were overrepresented in the low-consumption category, whereas male divers were more frequently classified within the moderate-consumption group. Adequate protein intake is increasingly recognized as important for maintaining muscle mass and function, particularly in physically active individuals. It contributes to recovery, training adaptation, and the preservation of functional capacity over time (21). This aspect may be especially relevant in freediving, and particularly in depth disciplines or dynamic apnea, where efficient propulsion, body control, and movement economy contribute to performance and safety (2, 3).

In this context, maintaining adequate muscle mass and functional strength may be relevant in freediving, particularly in disciplines involving propulsion and greater whole-body work, such as dynamic apnea and some depth disciplines (22). However, performance in these disciplines likely depends not only on muscle mass, but also on movement economy and technical efficiency. These factors may help reduce oxygen cost during underwater locomotion (2, 23).

The lower relative consumption of protein-rich foods observed among women may reflect broader sex-related differences in habitual food choices rather than sport-specific nutritional strategies alone. Such differences have been reported in both general and athletic populations and may be influenced by cultural, behavioral, or food preference factors (24, 25). However, the present study did not quantify total protein intake or assess body composition in relation to dietary intake; therefore, no conclusions can be drawn regarding the adequacy of protein intake in either sex.

No significant differences in habitual dietary patterns were observed across years of practice, suggesting that general food habits remained relatively stable regardless of freediving experience. This may indicate that progression within the sport is not necessarily accompanied by major long-term dietary changes, at least not at the level captured by the present food frequency questionnaire. Rather than modifying their overall dietary pattern, more experienced freedivers may be more likely to adopt acute or context-specific nutritional strategies surrounding training or competition, such as meal timing (26), hydration status, pre-apnea fasting (1), or nitrate supplementation (9, 10), which were not fully characterized in the present study.

Similarly, fasting practices did not differ significantly according to sex or years of practice. This finding is noteworthy given that fasting or training in a fasted state is sometimes discussed in apnea contexts as a potential strategy to enhance dive comfort or prolong breath-hold performance (1, 2, 9, 13). However, the absence of differences in the present sample suggests that such practices may reflect individual preference rather than a consistent behavior linked to sex or competitive experience. Overall, these findings suggest that habitual dietary patterns are relatively homogeneous among Spanish competitive freedivers. However, this does not exclude the possibility that specific dietary components may still influence physiological responses or hypoxia-related risk.

### 4.2 Occurrence of hypoxic events

Nearly one-third of divers reported experiencing at least one hypoxic event during their sporting careers, highlighting that such events are relatively frequent even among trained freedivers. This finding is consistent with the literature on adverse events in competitive freediving, which reports a non-negligible incidence of hypoxia-related events, particularly during high-performance attempts and competitive settings (7, 27).

No significant association was observed between sex and hypoxic events, suggesting that sex is not a primary determinant of susceptibility in competitive freediving. This is in line with previous research indicating that, although men typically present larger lung volumes, greater oxygen storage capacity, and larger spleen size, the key physiological responses to hypoxia–such as peripheral oxygen saturation (SpO₂) decline and activation of the diving reflex–do not differ substantially between sexes (28, 29). These anatomical and storage differences may influence performance duration (28), but they do not appear to affect the likelihood of experiencing hypoxic events.

In contrast, years of practice was significantly associated with hypoxic events, showing a clear gradient according to event severity. Beginners reported few or no events, whereas LMC was more frequently observed among divers with 1 to 4 years of experience, and more severe events, such as BO, predominated in those with 4 to 10 years of practice. This pattern suggests that the risk of hypoxic events may increase with experience, likely reflecting greater cumulative exposure to demanding apneas rather than inherent physiological vulnerability. Physiological evidence supports this interpretation, indicating that more challenging conditions–such as deeper or prolonged dives and higher work rates–lead to greater oxygen desaturation and increase the likelihood of a hypoxic event (30). Even when dive duration is similar, increased depth or physiological strain can exacerbate SpO₂ decline, thereby increasing the probability of reaching critically low oxygen levels (30). Overall, these results suggest that hypoxic events in competitive freediving are more strongly related to cumulative exposure to training and competition than to sex-related physiological differences.

### 4.3 Dietary habits and the occurrence of hypoxic events

The present results suggest that stimulant beverage consumption may be associated with the occurrence of hypoxic events in competitive freedivers, although this relationship appears to be complex. Notably, moderate consumption was associated with higher odds of reporting hypoxic events, whereas no significant association was observed for high consumption.

From a physiological perspective, caffeine may modulate autonomic and cardiovascular responses to apnea (31). These effects are particularly relevant in freediving, where bradycardia and peripheral vasoconstriction are key adaptive responses (32). In addition, caffeine may also increase oxygen consumption and the metabolic rate during hypoxic conditions (33). Conversely, habitual consumers may develop tolerance to these effects (34), which could partly account for the absence of association observed in the high-consumption group.

The non-linear pattern observed in this study, with moderate but not high consumption associated with hypoxic events, may also reflect behavioral and methodological factors. For instance, moderate consumption was the most prevalent category across competitive levels, which may increase the probability of detecting associations in this group. In addition, factors not captured in the present analysis–such as timing of intake, dosage, or individual sensitivity to stimulants, as well as other unmeasured physiological or behavioral factors (e.g., training intensity, sleep quality, and hydration status) may have influenced the observed results (34–36).

Importantly, these findings should be interpreted in the context of the primary results of this study, which identified years of practice as a consistent predictor of hypoxic events. However, this association should not be interpreted as causal. Within this framework, stimulant consumption may act as a secondary or interacting factor rather than an independent determinant. Nevertheless, the wide confidence intervals observed in the regression analyses indicate substantial uncertainty around effect estimates, suggesting that findings should be interpreted cautiously, particularly given the limited sample size and potential subgroup instability.

Several limitations should also be acknowledged. The cross-sectional design precludes causal inference. The predominance of male participants limited the feasibility of robust sex-specific analyses. Moreover, weekly training volume was not assessed in the present study. Although years of practice were included as a proxy of exposure, they may not fully capture actual training load or cumulative hypoxic exposure. Variables such as training frequency, session duration, and intensity would improve the characterization of exposure in future studies.

In addition, the relatively small sample size (*n* = 71) may have limited statistical power, particularly in subgroup analyses, and may constrain generalizability. However, this sample size is consistent with previous research in competitive freediving populations, where recruitment of larger cohorts is inherently challenging. Finally, the reliance on self-reported data may introduce recall bias.

Future studies incorporating larger and more balanced samples, objective physiological monitoring, and more detailed assessment of nutritional strategies–including timing, dosage, type of stimulant intake, and overall dietary adequacy–are warranted to further clarify the role of stimulant consumption in hypoxia-related risk in apnea sports.

## 5 Conclusion

The findings of the present study indicate that the risk of hypoxic events in competitive freediving is primarily related to exposure-dependent factors, particularly years of practice, which likely reflect cumulative training load and participation in more demanding apneas. In contrast, overall dietary habits did not appear to play a major independent role, within the limitations of the dietary assessment method, although stimulant beverage consumption may act as a potential modulatory factor under specific conditions. The observed non-linear association and the uncertainty surrounding the estimates highlight the complexity of this relationship and the need for cautious interpretation. Taken together, these findings emphasize the importance of considering both training-related exposure and contextual behavioral factors when evaluating hypoxia-related risk in competitive freediving.

## Data Availability

The raw data supporting the conclusions of this article are not publicly available to protect participant confidentiality and privacy. Requests to access the datasets should be directed to jsarola@tecnocampus.cat.
